# *tarsal-less* is expressed as a gap gene but has no gap gene phenotype in the moth midge *Clogmia albipunctata*

**DOI:** 10.1098/rsos.180458

**Published:** 2018-08-22

**Authors:** Eva Jiménez-Guri, Karl R. Wotton, Johannes Jaeger

**Affiliations:** 1 EMBL/CRG Research Unit in Systems Biology, Centre de Regulació Genòmica (CRG), The Barcelona Institute of Science and Technology (BIST), Dr. Aiguader 88, 08003 Barcelona, Spain; 2 Universitat Pompeu Fabra (UPF), Barcelona, Spain; 3 Centre for Ecology and Conservation, College of Life and Environmental Sciences, University of Exeter, Penryn, Cornwall TR10 9EZ, UK

**Keywords:** *tarsal-less* (*tal*), polycistronic gene, gap gene system, pattern formation, developmental evolution, *Clogmia albipunctata*

## Abstract

Gap genes are involved in segment determination during early development of the vinegar fly *Drosophila melanogaster* and other dipteran insects (flies, midges and mosquitoes). They are expressed in overlapping domains along the antero-posterior (A–P) axis of the blastoderm embryo. While gap domains cover the entire length of the A–P axis in *Drosophila,* there is a region in the blastoderm of the moth midge *Clogmia albipunctata*, which lacks canonical gap gene expression. Is a non-canonical gap gene functioning in this area? Here, we characterize *tarsal-less* (*tal*) in *C. albipunctata*. The homologue of *tal* in the flour beetle *Tribolium castaneum* (called *milles-pattes, mlpt*) is a *bona fide* gap gene. We find that *Ca-tal* is expressed in the region previously reported as lacking gap gene expression. Using RNA interference, we study the interaction of *Ca-tal* with gap genes. We show that *Ca-tal* is regulated by gap genes, but only has a very subtle effect on *tailless (Ca-tll),* while not affecting other gap genes at all. Moreover, cuticle phenotypes of *Ca-tal* depleted embryos do not show any gap phenotype. We conclude that *Ca-tal* is expressed and regulated like a gap gene, but does not function as a gap gene in *C. albipunctata*.

## 1. Introduction

The gap gene network provides the first layer of zygotic regulation in the segmentation gene hierarchy of dipteran insects (flies, midges and mosquitos). In the vinegar fly *Drosophila melanogaster*, this network consists of the trunk gap genes *hunchback (hb), Krüppel (Kr), knirps (kni)* and *giant (gt),* with additional inputs from the terminal gap genes *tailless (tll)* and *huckebein (hkb)* [1]. In other cyclorrhaphan flies, such as the hoverfly *Episyrphus balteatus* [2,3] and the scuttle fly *Megaselia abdita* [4–6]*,* gap gene expression and regulation is strongly conserved. It leads to a set of virtually identical expression domains, comprising overlapping regions of blastoderm nuclei/cells, at the onset of gastrulation. Outside the cyclorrhaphan clade, among the nematoceran Diptera, there is little functional evidence on gap gene regulation although expression patterns have been described in the malaria mosquito *Anopheles gambiae* [7].

Here, we focus on another emerging nematoceran model system, the moth midge *Clogmia albipunctata* (Diptera, Psychodidae). In this species, we have a detailed description of the spatial arrangement [8,9] as well as the temporal dynamics [10] of gap gene expression. This descriptive evidence reveals a region of the *C. albipunctata* blastoderm embryo which is not covered by expression of any gap gene known from *D. melanogaster* [9]. This region lies between the abdominal domain of the *C. albipunctata* homologue of *kni* and *knirps-related* (called *knirps-like, knl*) and the posterior terminal domain of *tll* [9,10]*.* It suggests that we may be missing a posterior gap gene in this species.

One candidate for this missing gap gene in *C. albipunctata* is *tarsal-less* (*tal*) [11]*,* also called *polished rice* (*pri*) [12]. *tal/pri* is a polycistronic gene encoding a long primary transcript from which several short peptides are produced that are required in different stages of embryonic development. It is part of a large class of polycistronic genes with small open reading frames (sORF/smORF), small encoded peptides or microproteins that play a wide range of roles in physiology, development and cell differentiation [13,14]. In *D. melanogaster, tal/pri* is first expressed in a stripe-like expression pattern at the late blastoderm stage [12] (by expression stripe, we mean a narrow expression domain, only a few nuclei wide). It is involved in epithelial morphogenesis and leg development [11,12,15–18], but has no role in early embryonic patterning or segment determination.

Interestingly, a homologue of *tal/pri* was first described in the flour beetle *Tribolium castaneum* under the name of *mille-pattes* (*mlpt*) [19]. By contrast to *tal/pri* in *D. melanogaster, mlpt* in *T. castaneum* has a segmentation function acting as a *bona fide* gap gene [19]. *mlpt* is expressed in a gap-like fashion, with an anterior and a posterior terminal domain at blastoderm stage; subsequently, the anterior domain resolves into two stripes, and the terminal domain retracts from the pole and shifts anteriorly over time during germband extension; a third posterior domain appears at this stage; finally, *mlpt* is expressed in the peripheral nervous system and the forming appendage joints at later stages of development, which is similar to its expression pattern in *D. melanogaster* [19]. Knock-down of *mlpt* in *T. castaneum* by RNA interference (RNAi) leads to a gap-like phenotype with missing abdominal segments [19]. *mlpt* regulates trunk gap genes *hb, Kr* and *gt,* and is itself regulated by *hb* and *Kr* [19].

Here, we characterize expression of *tal/pri* in *C. albipunctata,* and examine its interactions with other segmentation genes using RNAi knock-down assays. We show that it exhibits a gap-gene-like expression pattern at the blastoderm stage. As in *T. castaneum,* it is expressed in an anterior and a posterior terminal domain, which later split into narrow stripes. In contrast to *T. castaneum*, however, *tal/pri* does not regulate gap genes in *C. albipunctata,* with the possible exception of its interaction with the posterior terminal *tll* domain. Even though it is regulated by gap genes *hb, Kr* and *knl*, it does not exhibit any gap-like phenotype when knocked down. This evidence suggests that although *tal/pri* is expressed and regulated in a gap-gene-like manner, it cannot be classified as a *bona fide* gap gene in *C. albipunctata*.

## 2. Results and discussion

### 2.1. Characterization of *tarsal-less* in the moth midge *C. albipunctata*

We searched the early embryonic transcriptome of *C. albipunctata* [20] for a *tal* homologue using the *D. melanogaster* amino acid sequences for the small peptides encoded by *tal*. Our search identified a 2277 nt fragment that contained several short peptide repeats, probably corresponding to a primary transcript. Upon *in silico* translation, it was confirmed as a homologue of *tal/pri/mlpt* in *C. albipunctata*. We will call this fragment *Ca-tal*. Specific primers were generated to clone the gene from cDNA, and empirically confirm its sequence (see Material and methods). The *Ca-tal* sequence has been deposited in GenBank under accession number MG783326.

**Figure 1.**
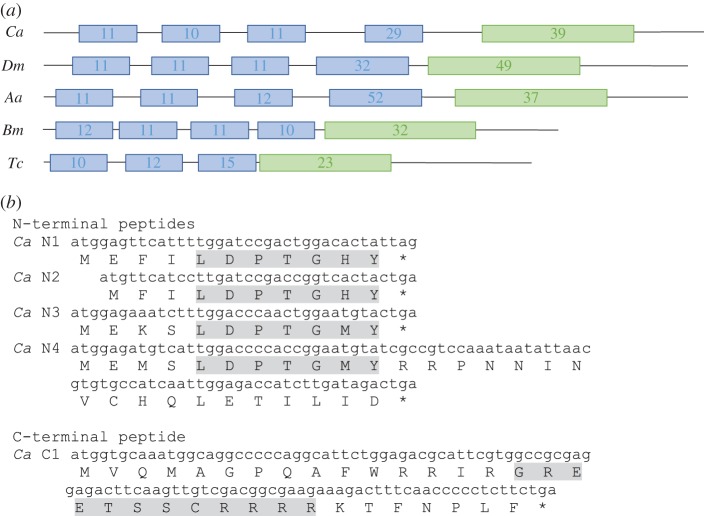
Sequence of *Ca-tal* and *in silico* translation. (*a*) Schematic comparisons of small ORFs in *tal/pri/mlpt* homologues of *C. albipunctata* (*Ca*), *D. melanogaster* (*Dm*), *Aedes aegypti* (*Aa*), *Bombyx mori* (*Bm*) and *T. castaneum* (*Tc*) (sequences as described in [19]). Blue boxes represent N-terminal peptides; green boxes represent C-terminal peptides. Numbers represent peptide length in amino acids. The fourth N-terminal peptide in *D. melanogaster* and *A. aegypti* has two conserved repeats. (*b*) Five conserved peptides are found in the transcriptomic sequence of *Ca-tal*, as retrieved from the Diptex database (CAL_comp2583_c0_seq1): four N-terminal (*Ca* N1–4), and one C-terminal peptide (*Ca* C1). Conserved consensus regions for each peptide types are highlighted in grey. Complete primary transcript sequence is deposited in GenBank with accession number MG783326.

The polycistronic sequence of *Ca-tal* shows general structural similarities to *tal* genes in other organisms (figure 1*a*). *tal* genes exhibit variable numbers of repeats of N-terminal peptides containing a consensus region of LDPTGXY, and one C-terminal peptide with the consensus domain GREETSSCRRRR [19]. In *Ca-tal,* we find four short repeated N-terminal peptides of 11, 10, 11 and 29 amino acids separated by 111, 126 and 334 nt length, respectively, plus a longer 39 amino acid C-terminal peptide separated from the closest N-terminal peptide by 348 nt (figure 1*b*).

### 2.2. Temporal expression profile of *Ca-tal* in the embryo

**Figure 2.**
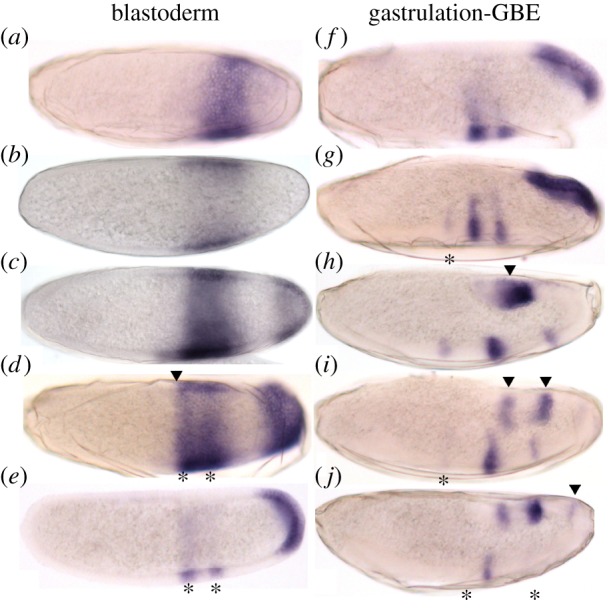
Expression pattern of *Ca-tal* during early embryo development of *C. albipunctata*. Colorimetric (enzymatic) ISH against *Ca-tal* (blue) in embryos ranging from early cellular blastoderm (*a*) to gastrulation (*f*), and germ band elongation (GBE) (*g*–*j*). Time increases downwards in each column. Embryos oriented laterally: anterior is to the left, dorsal to the top. See text for details.

We have characterized the expression pattern of *Ca-tal* in the embryo of *C. albipunctata* from the blastoderm up to the extended germband stage [21] using enzymatic (colorimetric) *in situ* hybridization (ISH) (figure 2). The earliest pattern we detect is a posterior expression domain in the trunk region of the blastoderm embryo, covering 65–80% antero-posterior (A–P) position (figure 2*a*). This domain shifts anteriorly over time (figure 2*b*). By the time it has reached 55–75% A–P position, a second terminal domain becomes apparent at the posterior pole (figure 2*c*). Both domains continue to shift and expand anteriorly (figure 2*d*), consistent with shifts observed for posterior gap genes during the blastoderm stage [9,10]. Before gastrulation, the anterior border of the more anterior *Ca-tal* domain reaches 55% A–P position (figure 2*d*, arrowhead), and this domain starts to split into two stripes (figure 2*d*, asterisks). By the same time, the posterior terminal domain has expanded to 85% A–P position.

By the onset of gastrulation, the anterior domain has resolved completely into two stripes (figure 2*e*, asterisks). A weak third stripe appears shortly thereafter in a more anterior position (figure 2*g*, asterisk). This dynamic pattern is similar to what has been reported for the *tal* homologue *mille-pattes (mlpt)* in the flour beetle *T. castaneum* [19]. The terminal domain follows the morphogenetic movement of the posterior pole region during gastrulation [21], moving to the dorsal side of the embryo (figure 2*f–h*); at the same time, this domain clears from the pole (figure 2*h*, arrowhead) and divides into two sub-terminal stripes (figure 2*i*, arrowheads). During germband elongation, the first, and later the third, stripe of the anterior domain fade away (figure 2*i,j*, asterisks). Finally, an additional stripe appears anterior of the two sub-terminal stripes (figure 2*j*, arrowhead).

**Figure 3.**
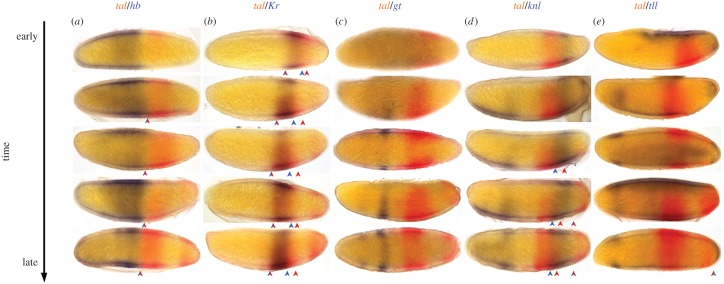
Relative localization of *Ca-tal* and gap gene expression in *C. albipunctata* blastoderm embryos. Colorimetric (enzymatic) ISH against *Ca-tal* (red) is shown with RNA patterns of gap genes (in blue) during the blastoderm stage. Stains as indicated by column headings: (*a*) *tal* (red)/*hb* (blue); (*b*) *tal* (red)/*Kr* (blue); (*c*) *tal* (red)/*gt* (blue); (*d*) *tal* (red)/*knl* (blue); (*e*) *tal* (red)/*tll* (blue). Arrowheads point at domain boundaries for clarification (red, tal boundary; blue, gap gene boundary; red and blue, coinciding gap-tal boundaries). Time increases downwards. Embryos oriented laterally: anterior is to the left, dorsal to the top.

As a next step, we performed double *in situ* hybridizations to define the expression of *Ca-tal* in reference to the gap gene domains in the *C. albipunctata* blastoderm (figure 3). The anterior border of the more anterior *Ca-tal* domain coincides with the posterior border of the anterior domain of *Ca-hb* (figure 3*a*, double colour arrowhead), both shifting anteriorly in concert over time. The central domain of *Ca-Kr* and the more anterior *Ca-tal* domain show extensive overlap (figure 3*b*, double colour arrowhead), although the latter extends slightly further posterior (figure 3*b*, single colour arrowheads). Domains of *Ca-gt* and *Ca-tal* never overlap and are positioned far from each other in the embryo (figure 3*c*). This is because *C. albipunctata* lacks a posterior *gt* domain, unlike *D. melanogaster* [9]. The more anterior domain of *Ca-tal* and the abdominal domain of *Ca-knl* overlap in the anterior region of the latter (figure 3*d*, single colour arrowheads). The posterior border of the abdominal *Ca-knl* domain coincides with the anterior border of the terminal *Ca-tal* domain (figure 3*d*, double colour arrowhead). The posterior terminal *Ca-tll* domain overlaps with the posterior part of the terminal *Ca-tal* domain (figure 3*e*). In our double *in situs*, we see that the terminal *Ca-tal* domain already clears from the posterior pole during the blastoderm stage, so that its posterior boundary comes to coincide with the anterior border of *Ca-tll* just before the onset of gastrulation (figure 3*e*, double colour arrowhead).

Our results show that *Ca-tal* is expressed in a gap-gene-like manner during the blastoderm stage, partially overlapping with previously characterized gap domains in *C. albipunctata* [9]. Intriguingly, the terminal *Ca-tal* domain covers a region of the *C. albipunctata* blastoderm—between the abdominal *Ca-knl* domain and the terminal domain of *Ca-tll*—in which no gap gene expression has been detected before [9,10]. In contrast, *tal* is not expressed like a gap gene in *D. melanogaster*, where its transcripts appear directly in a stripe-like pattern during the late blastoderm stage [12]. Early *Ca-tal* expression shows much more resemblance to that of its homologue *mlpt* in *T. castaneum,* which acts as a *bona fide* gap gene in that species [19]. This suggests that *Ca-tal* may also play the role of a gap gene in *C. albipunctata.* In order to test this possibility, we performed knock-down by RNAi of *Ca-tal, Ca-tll,* and other trunk gap genes.

### 2.3. *Ca-tal* does not regulate, but is regulated by trunk gap genes

To assess the effect of *Ca-tal* on the gap genes in *C. albipunctata,* we performed RNAi knock-down against *Ca-tal* following a previously published protocol [22]. The resulting *tal-*depleted embryos were stained by colorimetric ISH for trunk gap genes *Ca-hb*, *Ca-Kr*, Ca-*gt* and *Ca-knl*, as well as the terminal gap gene *Ca-tll.* The other terminal gap gene, *huckebein* (*hkb*), is not expressed at the relevant stages in *C. albipunctata* [9]. We do not observe any clearly detectable differences in the expression patterns of the trunk gap genes in *Ca-tal* knock-down embryos (electronic supplementary material, figure S1). Quantitative assessment of domain boundary positions using our FlyGUI/FlyAGE image-processing pipeline [10,23] does not reveal any significant differences to the wild-type either (not shown). The only potential effect of *Ca-tal* on gap genes is the reduced expression in the posterior terminal domain of *Ca-tll* in a small percentage of *Ca-tal* knock-down embryos (4 out of 17; electronic supplementary material, figure S1*e*,*f*,*k*,*l*). Target genes further downstream in the segmentation gene cascade, such as the pair-rule gene *even-skipped (eve)*, and the segment polarity genes *wingless (wg)* and *engrailed (en)*, also fail to show any clearly detectable defects in *Ca-tal* knock-down embryos (not shown). This suggests that *Ca-tal* does not play any essential role in segmentation gene regulation in *C. albipunctata.*

**Figure 4.**
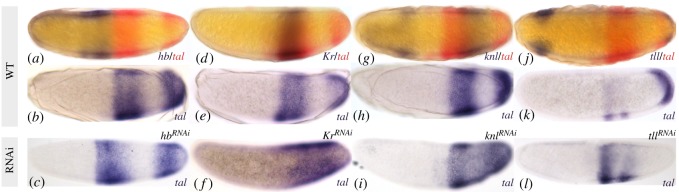
Effect of gap gene depletion by RNAi on *Ca-tal* expression. Colorimetric (enzymatic) ISH against *Ca-tal* and gap genes in *C. albipunctata* blastoderm embryos. (*a*,*d*,*g*,*j*) Wild-type (WT) embryos stained against *Ca-tal* (red) and gap genes (blue) as indicated in the lower right corner of each panel. (*b*,*e*,*h*,*k*) Wild-type (WT) embryos stained against *Ca-tal* only (blue). (*c*,*f*,*i*,*l*) *Ca-tal* expression (blue) in embryos depleted for gap RNA as indicated in the top right corner of each panel. WT panels provided as ready comparison to gap-depleted embryos. Embryos oriented laterally: anterior is to the left, dorsal to the top.

Next, we investigated whether *Ca-tal* is regulated by gap genes. We assayed *Ca-tal* expression in embryos treated with RNAi against *Ca-hb*, *Ca-Kr*, *Ca-gt*, *Ca-knl* and *Ca-tll* using colorimetric ISH (figure 4). The expression pattern of *Ca-tal* was affected by all gap genes with the exception of *Ca-gt* (not shown). In blastoderm embryos depleted of *Ca-hb*, the more anterior domain of *Ca-tal* is displaced anteriorly, extending past 45% A–P position (figure 4*c*; 10 out of 28 embryos). This suggests that *Ca-hb* positions the anterior border of expression of *Ca-tal* through repression. Alternatively, this repression could be indirect, mediated through repression of the activator encoded by *Ca-Kr* in this region (see below). In embryos depleted of *Ca-Kr*, we observe a loss of the more anterior *Ca-tal* domain, while its terminal domain appears to expand anteriorly (figure 4*f*, 17 out of 20 embryos). This is consistent with a dual influence of *Ca-Kr,* with an activating effect on the more anterior domain, and repression on the terminal domain of *Ca-tal*. However, it is not clear whether both of these effects are direct. Activation could be mediated through repression of repressor *Ca-knl* by *Ca-Kr*. This is unlikely, as *Ca-knl* is not affected in knock-downs of *Ca-Kr* (electronic supplementary material, figure S2, 17 out of 17 embryos). Still, we cannot exclude indirect activation mediated through repression of another unknown repressor. Finally, the effect of *Ca-Kr* could be interpreted as a deletion of the region between the two *Ca-tal* domains. This, however, seems unlikely, because *Ca-Kr* is not expressed near the potentially affected region of the embryo (cf. Figure 3*b*) and *Ca-knl* is still expressed there in *Ca-Kr* RNAi-treated embryos (electronic supplementary figure S2*b*). In embryos depleted of *Ca-knl,* we see strong ectopic expression of *Ca-tal* between its two domains of expression at the blastoderm stage (figure 4*i*, 25 out of 54 embryos). This suggests repression *of Ca-tal* by *Ca-knl*. The effect is probably weak, because the de-repression seen in figure 4*i* is incomplete, and the expression patterns of *Ca-tal* and *Ca-knl* show extensive overlap in the wild-type (figure 3*d*). Just as in the case of *Ca-hb* knock-downs discussed above, this effect could be indirect, mediated through *Ca-Kr*. In late blastoderm embryos depleted of *Ca-tll*, the terminal domain of *Ca-tal* expression is either completely abolished or strongly reduced (figure 4*l*, 17 out of 44 embryos). Taken together, our evidence suggests that *Ca-tal* and *Ca-tll* activate each other in *C. albipunctata*.

### 2.4. *Ca-tal* and *Ca-gt* do not exhibit gap gene phenotypes in *C. albipunctata*

**Figure 5.**
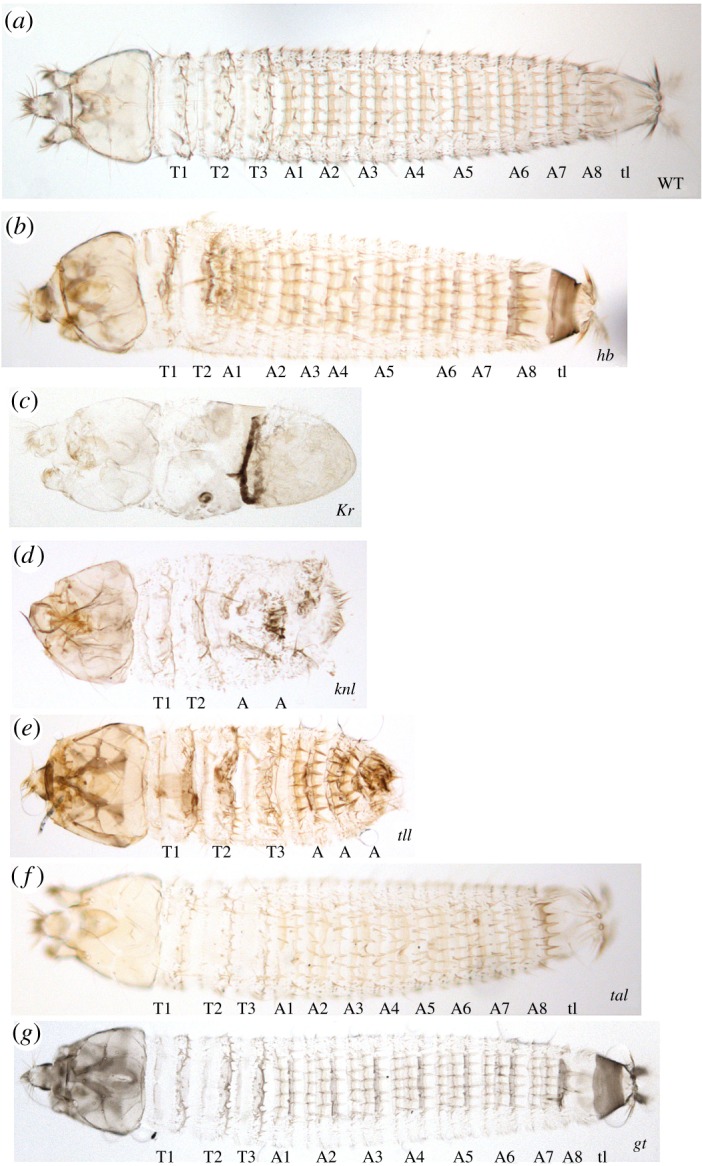
Cuticle preparations of late-stage wild-type and RNA-depleted embryos. (*a*) Wild type, (*b*) *Ca-hb* RNAi, (*c*) *Ca-Kr* RNAi, (*d*) *Ca-knl* RNAi, (*e*) *Ca-tll* RNAi, (*f*) *Ca-tal* RNAi and (*g*) *Ca-gt* RNAi. Labels indicate thoracic (T) and abdominal (A) segments, as well as the telson (tl). Wild-type cuticles include three thoracic segments, eight abdominal segments, and the telson (*a*). Segment numbers in depleted cuticles do not necessarily indicate exact segment identity, but reflect the number of segments present in each knock-down embryo. Cuticles shown in dorsal view, anterior is to the left. See Material and methods for details on preparation.

To further examine the function of *Ca-tal* and the gap genes in *C. albipunctata*, we obtained cuticle preparations of late-stage wild-type and RNAi embryos according to a previously published protocol (figure 5) [22]. In cuticles of embryos treated with RNAi against *Ca-hb* (*n* = 22), we observed a reduction in the number of thoracic segments in all specimens: seven embryos showed no, nine embryos one and six embryos two remaining thoracic segments (figure 5*b*). We only managed to obtain two cuticles of embryos treated with RNAi against *Ca-Kr.* Both of them exhibit general A–P polarity, but no thoracic or abdominal segments are discernible (figure 5*c*). Similarly, severe defects were observed in the two cuticles we obtained from embryos treated with RNAi against *Ca-knl*: these embryos show two recognizable thoracic and one or two abdominal segments, albeit with severe dorsal defects, as well as an abnormal posterior terminal region (figure 5*d*). Cuticles of embryos treated with RNAi against *Ca-tll* (*n* = 15) show a much less penetrant phenotype. In four individuals, the telson is missing, and two show a severe reduction of the number of abdominal segments (figure 5*e*). Only one specimen exhibited defects in the head and the thoracic region (not shown). We could detect no gap gene phenotypes or other obvious and consistent segmentation defects in embryos depleted for *Ca-tal* and *Ca-gt* (figure 5*f*,*g*)*.* However, in 2 out of 39 of the *gt* and 5 out of 55 of the *tal* depleted cuticles we observe small hemilateral abnormalities (electronic supplementary material, figure S3 A, B, asterisks). We cannot rule out a weak effect of RNA depletion, but the cause of these abnormalities could also be mechanical or unspecific. We do not observe this type of effect in the other RNAi injected cuticles. The evidence from our cuticle preparations suggest that *hb, Kr, kni/knl* and *tll* have conserved roles as gap genes in *C. albipunctata,* while *gt* and *tal* are expressed in a gap-like manner (*tal* also being regulated by other gap genes) but do not play a classical gap-like role in trunk segment determination in this species.

## 3. Conclusion

We have characterized the homologue of *tal/pri/mlpt* in the nematoceran moth midge *C. albipunctata*. Similar to its homologues in other organisms [11,19], it produces a polycistronic primary transcript, which codes for several short peptides. We have shown that *Ca-tal* is expressed in a gap-gene-like manner in *C. albipunctata,* unlike in *D. melanogaster* where it initiates transcription in refined stripes during the late blastoderm stage [12]. We show that these early stages of expression are regulated by gap genes in *C. albipunctata.* Later expression patterns are more conserved between the two species. Despite its suggestive early embryonic expression pattern, *Ca-tal* cannot be classified as a segmentation gene. Our evidence reveals that *Ca-tal* is not regulating other segmentation genes, and does not cause a gap-like or any other segmentation phenotype upon knock-down by RNAi.

**Figure 6.**
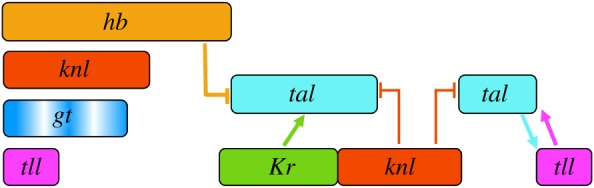
Schematic summary of *Ca-tal* expression and regulation. Coloured boxes indicate the relative position of expression domains along the A–P axis (anterior is to the left, posterior to the right). Arrows indicate activation, T-bars indicate repression, as suggested by our RNAi knock-down experiments.

The gap-like expression pattern of *Ca-tal* shows striking similarities to its homologue, the gap gene *mlpt* in the flour beetle *T. castaneum.* However, even this similarity may be superficial, as there are significant differences between the regulation of both homologues. The anterior domain of *Ca-tal* is repressed by Hb, while the posterior terminal domain is not affected in *hb* RNAi knock-downs (summarized in figure 6). In *T. castaneum*, the opposite is true: while the anterior domain of *mlpt* is not affected, the posterior domain forms late in if *hb* is depleted [19]. Furthermore, *Ca-tal* is repressed by *knl* (figure 6), while *kni* does not affect *mlpt* expression in *T. castaneum* [19]. In contrast, *mlpt* is activated by *gt* [19], while *Ca-tal* and *gt* show no genetic interaction. The only similarity between the two species is the role of *Kr* in *tal/mlpt* regulation: ectopic expression is seen upon *Kr* knock-down in the posterior of blastoderm embryos in *C. albipunctata* and *T. castaneum.* Based on the available evidence, it remains unclear whether the early gap-like expression pattern of *tal/mlpt* is an ancestral feature of segmentation patterning, or whether it has evolved convergently in beetles and nematoceran dipterans. Functional data from other basally branching dipteran lineages or suitable outgroups will be required to resolve this outstanding question.

## 4. Material and methods

### 4.1. Gene identification, cloning and synthesis of RNA constructs

We searched the early embryonic transcriptome of *C. albipunctata* ([20]; http://diptex.crg.es) using peptide sequences from *D. melanogaster tarsal-less* retrieved from GenBank. A 2277 nt clone was obtained (Diptex clone CAL_comp2583_c0_seq1). This sequence was confirmed via PCR on *C. albipunctata* cDNA, and is deposited in GenBank under accession number MG783326. A 1.2 kb fragment of *Ca-tal*, containing all small ORFs, was used to obtain riboprobes for whole-mount ISH, as well as template for double-stranded RNA. For the gap genes, clones were the same as used in [9] (fragment size in parentheses): *Ca-hb,* AJ131041.1 (1800 nt); *Ca-Kr,* GU137323.1 (1200 nt); *Ca-knl,* GU137321.1 (800 nt); *Ca-gt,* GU137318.1 (1100 nt); *Ca-tll*, GU137320.1 (1400 nt). Fragments were cloned into the PCRII-TOPO vector (Invitrogen), and used to make DIG or FITC-labelled riboprobes for whole-mount ISH, as well as double-stranded RNA. RNAi constructs were synthesized as described in [22].

### 4.2. Embryo collection and fixation

Wild-type and RNAi-treated embryos of *C. albipunctata* were collected at blastoderm and post-gastrulation stages as described previously in [9]. Embryos were heat-fixed using a protocol adapted from [24]. In brief, embryos were dechorionated at the desired developmental stage by immersing them in 25% bleach for 45 s. Embryos where then dipped in boiling fixing solution (0.7% NaCl; 0.05% Tween20) for 20s. Heat fixation was stopped by adding room-temperature (RT) water to the solution. Embryos were subsequently post-fixed in formaldehyde (5%) and PBS/methanol. Devitellinization is achieved by vigorous shaking for 20 s in 50% heptane-methanol. Embryos were preserved in methanol. For RNAi-treatment, embryos were dechorionated manually using tungsten needles and fixed as described for wild-type.

### 4.3. Whole-mount *in situ* hybridization

Whole-mount ISH was performed as described for *C. albipunctata* in [22] and references therein. In brief, embryos were permeabilized after rehydration using Proteinase K (8 mg ml^−1^ PBT) for 7 min at RT, followed by post-fixation in 5% formaldehyde/PBT for 25 min. Overnight hybridization at 56°C was carried out with a labelled probe at a concentration of 0.5–1 ng µl^−1^. For detection, antibodies (anti-digoxigenin or fluorescein conjugated with alkaline phosphatase (Sigma)) were used at 1 : 2000 for 2 h. Staining was achieved using NBT/BCIP (blue) or fast red. Embryos were counterstained with DAPI, and mounted in 70% glycerol.

### 4.4. RNA interference

RNAi treatment was carried out based on protocols established in other dipteran species [4,5,25] as described previously in [22]. In brief, embryos were allowed to develop for 2 h before immersing them in 25% bleach for 10 s to weaken the chorion, then rinsed under tap water for 1 min. Embryos where aligned on a microscope slide against a glass capillary (Hilgenberg 1421602, 65 mm × OD 0.25 mm) and covered with a 3 : 1 mixture of 10 : 27 halocarbon oil. Aluminosilicate (rather than borosilicate) capillaries (pulled in Sutter P-97 Flaming/Brown Micropipette Puller) were used for the injections, maintaining a constant flow of liquid to avoid blocking of the needle. After injection embryos were allowed to develop for 7 h before being fixed and collected as previously described [24]. Buffer-only injections were used as a negative control, along with ISH staining for depleted transcripts. Cuticle preparations were performed as described in [22]. Only cuticles injected with double-stranded RNA presented abnormal phenotypes. Double-stranded RNA was injected at the following concentration: *Ca-hb*, 5.1 μM; *Ca-gt*: 5.9 and 3.8 µM; *Ca-Kr*: 5.4 µM; *Ca-knl*: 5.1 µM, *Ca-tal*: 7.9 and 4.2 µM; Ca-*tll*: 3.4 µM.

## Supplementary Material

Effect of Ca-tal depletion by RNAi on gap gene expression

## Supplementary Material

Lack of effect of Ca-Kr depletion on Ca-knl expression

## Supplementary Material

Cuticle preparations of late-stage RNA-depleted embryos for Ca-gt RNAi and Ca-tal RNAi

## Data accessibility

All embryo images are available from superfly.crg.eu.

## References

[1] Jaeger J 2011 The gap gene network. Cell. Mol. Life Sci. 68, 243–274. (10.1007/s00018-010-0536-y) 20927566 PMC3016493

[2] Lemke S, Schmidt-Ott U 2009 Evidence for a composite anterior determinant in the hover fly *Episyrphus balteatus* (Syrphidae), a cyclorrhaphan fly with an anterodorsal serosa anlage. Development 136, 117–127. (10.1242/dev.033530) 19060334

[3] Lemke S, Busch SE, Antonopoulos DA, Meyer F, Domanus MH, Schmidt-Ott U 2010 Maternal activation of gap genes in the hover fly *Episyrphus*. Development 137, 1709–1719. (10.1242/dev.055558) 20430746

[4] Stauber M, Taubert H, Schmidt-Ott U 2000 Function of *bicoid* and *hunchback* homologs in the basal cyclorrhaphan fly *Megaselia* (Phoridae). Proc. Natl Acad. Sci. USA 97, 10 844–10 849. (10.1073/pnas.190095397) PMC27111 10995461

[5] Lemke S, Stauber M, Shaw PJ, Rafiqi AM, Prell A, Schmidt-Ott U 2008 *bicoid* occurrence and Bicoid-dependent *hunchback* regulation in lower cyclorrhaphan flies. Evol. Dev. 10, 413–420. (10.1111/j.1525-142X.2008.00252.x) 18638318

[6] Wotton KR, Jiménez-Guri E, Crombach A, Janssens H, Alcaine-Colet A, Lemke S, Schmidt-Ott U, Jaeger J 2015 Quantitative system drift compensates for altered maternal inputs to the gap gene network of the scuttle fly *Megaselia abdita*. Elife 4, e04785 (10.7554/eLife.04785) PMC4337606 25560971

[7] Goltsev Y, Hsiong W, Lanzaro G, Levine M 2004 Different combinations of gap repressors for common stripes in *Anopheles* and *Drosophila* embryos. Dev. Biol. 275, 435–446. (10.1016/j.ydbio.2004.08.021) 15501229

[8] Rohr KB, Tautz D, Sander K 1999 Segmentation gene expression in the mothmidge *Clogmia albipunctata* (Diptera, Psychodidae) and other primitive dipterans. Dev. Genes Evol. 209, 145–154. (10.1007/s004270050238) 10079357

[9] García-Solache M, Jaeger J, Akam M 2010 A systematic analysis of the gap gene system in the moth midge *Clogmia albipunctata*. Dev. Biol. 344, 306–318. (10.1016/j.ydbio.2010.04.019) 20433825

[10] Crombach A, García-Solache MA, Jaeger J 2014 Evolution of early development in dipterans: reverse-engineering the gap gene network in the moth midge *Clogmia albipunctata* (Psychodidae). Biosystems 123, 74–85. (10.1016/j.biosystems.2014.06.003) 24911671

[11] Galindo MI, Pueyo JI, Fouix S, Bishop SA, Couso JP 2007 Peptides encoded by short ORFs control development and define a new eukaryotic gene family. PLoS Biol. 5, e106 (10.1371/journal.pbio.0050106) 17439302 PMC1852585

[12] Kondo T, Hashimoto Y, Kato K, Inagaki S, Hayashi S, Kageyama Y 2007 Small peptide regulators of actin-based cell morphogenesis encoded by a polycistronic mRNA. Nat. Cell Biol. 9, 660–665. (10.1038/ncb1595) 17486114

[13] Andrews SJ, Rothnagel JA 2014 Emerging evidence for functional peptides encoded by short open reading frames. Nat. Rev. Genet. 15, 193–204. (10.1038/nrg3520) 24514441

[14] Plaza S, Menschaert G, Payre F 2017 In search of lost small peptides. Annu. Rev. Cell Dev. Biol. 33, 391–416. (10.1146/annurev-cellbio-100616-060516) 28759257

[15] Pueyo JI, Couso JP 2008 The 11-aminoacid long Tarsal-less peptides trigger a cell signal in *Drosophila* leg development. Dev. Biol. 324, 192–201. (10.1016/j.ydbio.2008.08.025) 18801356

[16] Pueyo JI, Couso JP 2011 Tarsal-less peptides control Notch signalling through the Shavenbaby transcription factor. Dev. Biol. 355, 183–193. (10.1016/j.ydbio.2011.03.033) 21527259 PMC3940869

[17] Zanet J, Benrabah E, Li T, Pelissier-Monier A, Chanut-Delalande H, Ronsin B, Bellen HJ, Payre F, Plaza S 2015 Pri sORF peptides induce selective proteasome-mediated protein processing. Science 349, 1356–1358. (10.1126/science.aac5677) 26383956

[18] Kondo T, Plaza S, Zanet J, Benrabah E, Valenti P, Hashimoto Y, Kobayashi S, Payre F, Kageyama Y 2010 Small peptides switch the transcriptional activity of Shavenbaby during *Drosophila* embryogenesis. Science 329, 336–339. (10.1126/science.1188158) 20647469

[19] Savard J, Marques-Souza H, Aranda M, Tautz D 2006 A segmentation gene in *Tribolium* produces a polycistronic mRNA that codes for multiple conserved peptides. Cell 126, 559–569. (10.1016/j.cell.2006.05.053) 16901788

[20] Jiménez-Guri E, Huerta-Cepas J, Cozzuto L, Wotton KR, Kang H, Himmelbauer H, Roma G, Gabaldón T, Jaeger J 2013 Comparative transcriptomics of early dipteran development. BMC Genomics 14, 123 (10.1186/1471-2164-14-123) 23432914 PMC3616871

[21] Jiménez-Guri E, Wotton KR, Gavilán B, Jaeger J 2014 A staging scheme for the development of the moth midge *Clogmia albipunctata*. PLoS ONE 9, e84422 (10.1371/journal.pone.0084422) 24409296 PMC3883683

[22] Wotton KR, Alcaine-Colet A, Jaeger J, Jiménez-Guri E 2017 Non-canonical dorsoventral patterning in the moth midge *Clogmia albipunctata*. Evodevo 8, 20 (10.1186/s13227-017-0083-9) 29158889 PMC5683363

[23] Crombach A, Cicin-Sain D, Wotton KR, Jaeger J 2012 Medium-throughput processing of whole mount in situ hybridisation experiments into gene expression domains. PLoS ONE 7, e46658 (10.1371/journal.pone.0046658) 23029561 PMC3460907

[24] Rafiqi AM, Lemke S, Schmidt-Ott U 2011 *Megaselia abdita*: fixing and devitellinizing embryos. Cold Spring Harb. Protoc. 2011, 5602 (10.1101/pdb.prot5602) 21460051

[25] Rafiqi AM, Lemke S, Schmidt-Ott U 2011 *Megaselia abdita*: preparing embryos for injection. Cold Spring Harb. Protoc. 2011, 5601 (10.1101/pdb.prot5601) 21460050

