# Supplementary figures and images for: tarsal-less is expressed as a gap gene but has no gap gene phenotype in the moth midge Clogmia albipunctata
Source: R Soc Open Sci. 2018 Aug 22;5(8):180458. doi: 10.1098/rsos.180458 (PMC6124123; doi:10.1098/rsos.180458)

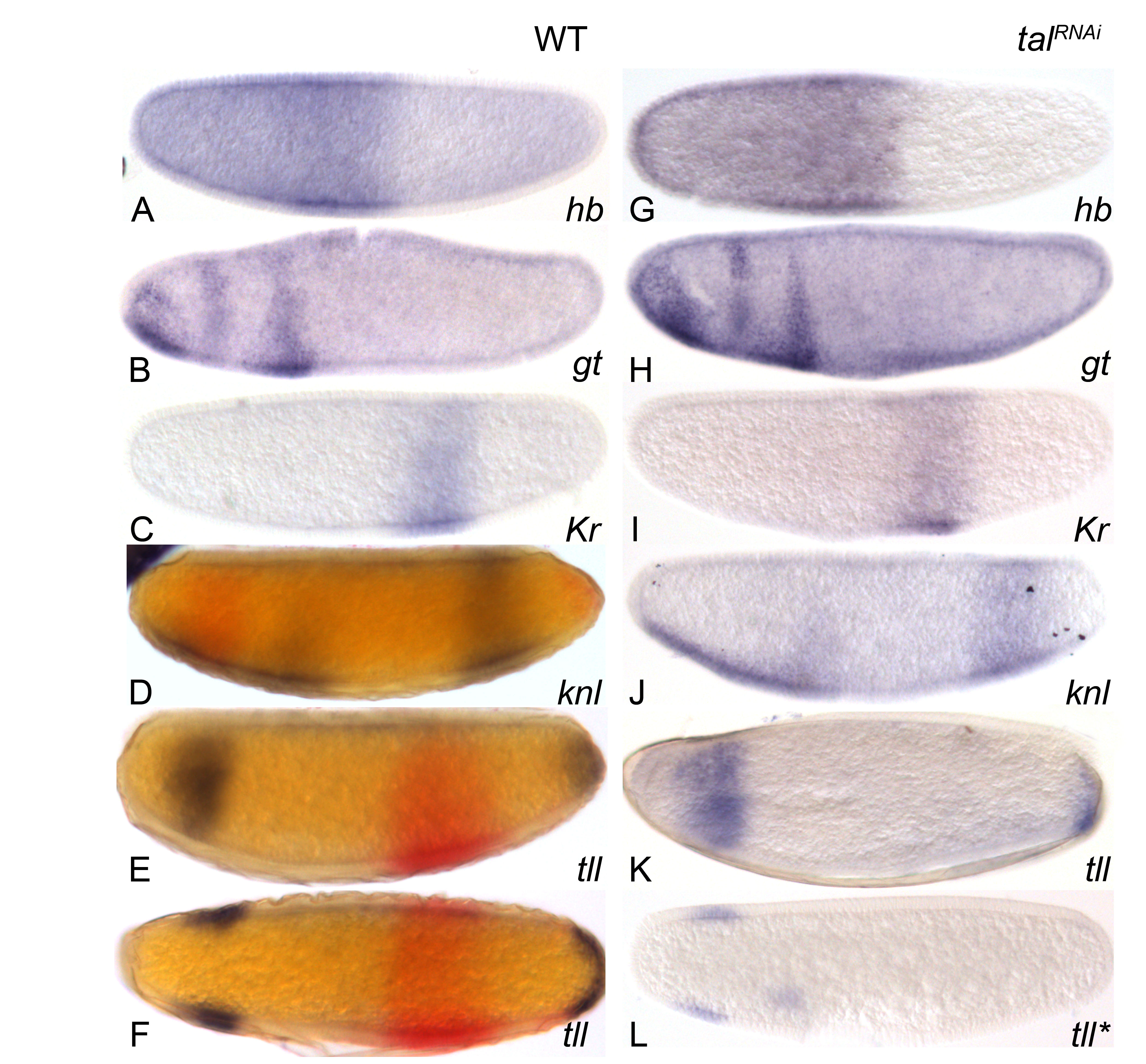

Supplement: Effect of Ca-tal depletion by RNAi on gap gene expression [file rsos180458supp1.jpg]

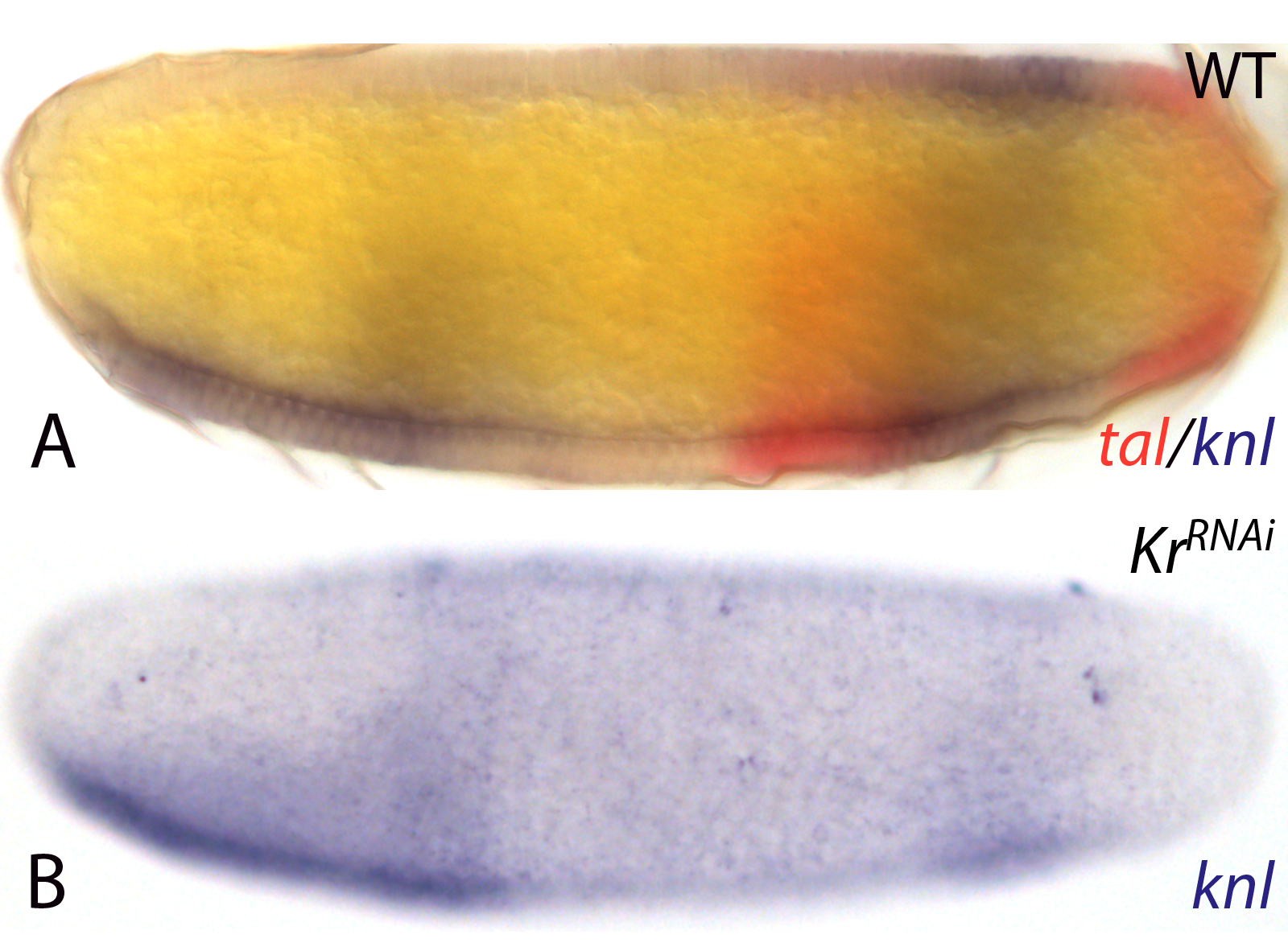

Supplement: Lack of effect of Ca-Kr depletion on Ca-knl expression [file rsos180458supp2.tif]

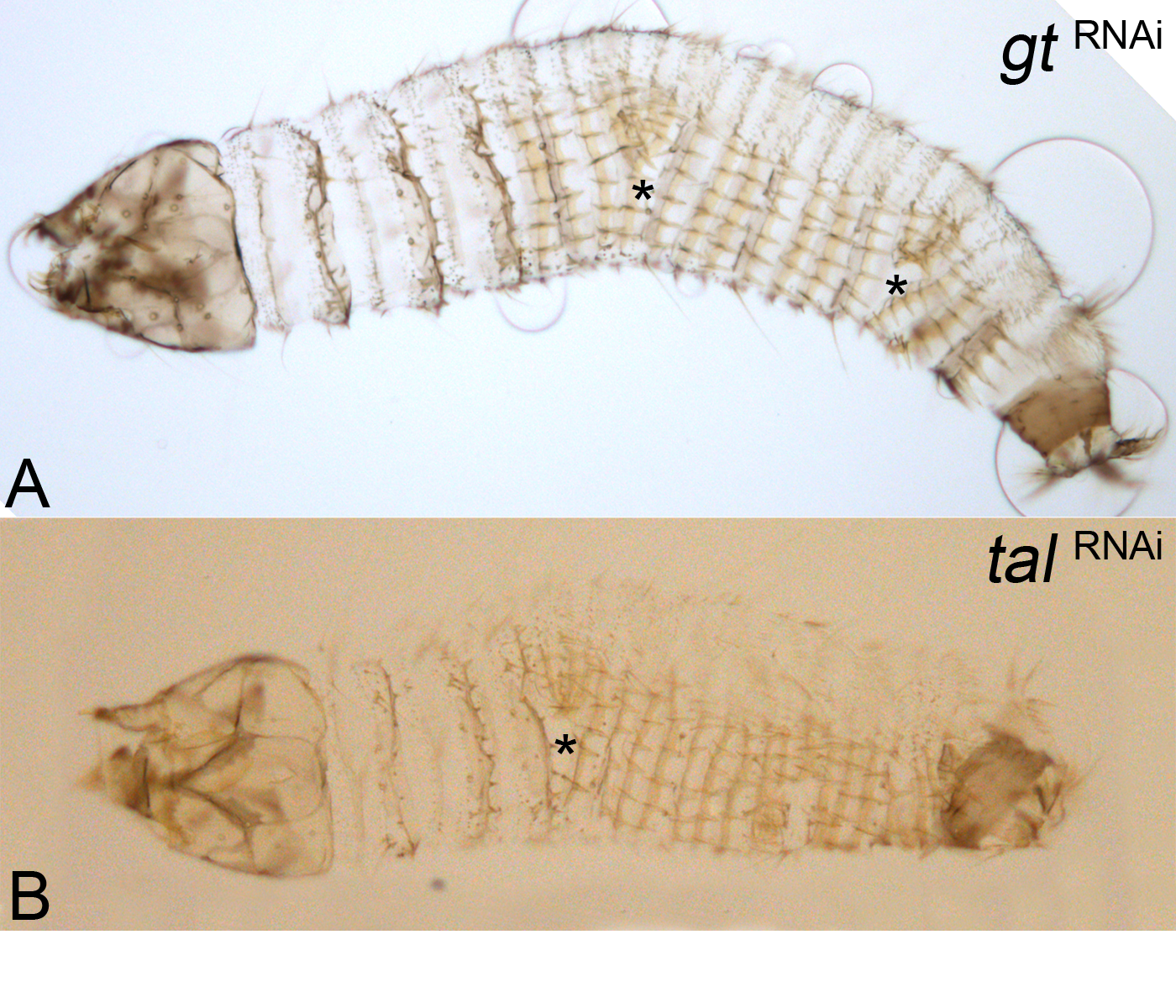

Supplement: Cuticle preparations of late-stage RNA-depleted embryos for Ca-gt RNAi and Ca-tal RNAi [file rsos180458supp3.tif]
